# 7-T MRI for brain virtual autopsy: a proof of concept in comparison to 3-T MRI and CT

**DOI:** 10.1186/s41747-020-00198-7

**Published:** 2021-01-14

**Authors:** Dominic Gascho, Niklaus Zoelch, Stefan Sommer, Carlo Tappero, Michael J. Thali, Eva Deininger-Czermak

**Affiliations:** 1 Department of Forensic Medicine and Imaging, Institute of Forensic Medicine, University of Zurich, Winterthurerstrasse 190/52, CH-8057 Zurich, Switzerland; 2 Department of Psychiatry, Psychotherapy and Psychosomatics, Hospital of Psychiatry, University of Zurich, Zurich, Switzerland; 3 Siemens Healthcare AG, Zurich, Switzerland; 4 Swiss Center for Musculoskeletal Imaging (SCMI), Balgrist Campus AG, Zurich, Switzerland; 5 Department of Radiology, Hôpital Fribourgeois, Villars-sur-Glâne, Switzerland; 6 Institute of Diagnostic and Interventional Radiology, University Hospital Zurich, Zurich, Switzerland

**Keywords:** Autopsy, Brain injuries (traumatic), Contrecoup injury, Magnetic resonance imaging, Tomography (x-ray, computed)

## Abstract

The detection and assessment of cerebral lesions and traumatic brain injuries are of particular interest in forensic investigations in order to differentiate between natural and traumatic deaths and to reconstruct the course of events in case of traumatic deaths. For this purpose, computed tomography (CT) and magnetic resonance imaging (MRI) are applied to supplement autopsy (traumatic death) or to supplant autopsy (natural deaths). This approach is termed “virtual autopsy.” The value of this approach increases as more microlesions and traumatic brain injuries are detected and assessed. Focusing on these findings, this article describes the examination of two decedents using CT, 3-T, and 7-T MRI. The main question asked was whether there is a benefit in using 7-T over 3-T MRI. To answer this question, the 3-T and 7-T images were graded regarding the detectability and the assessability of coup/contrecoup injuries and microlesions using 3-point Likert scales. While CT missed these findings, they were detectable on 3-T and 7-T MRI. However, the 3-T images appeared blurry in direct comparison with the 7-T images; thus, the detectability and assessability of small findings were hampered on 3-T MRI. The potential benefit of 7-T over 3-T MRI is discussed.

## Key points

- Brain virtual autopsy by computed tomography (CT) or magnetic resonance imaging (MRI) is an emerging approach in forensic medicine.
- In two decedents, CT missed brain coup/contrecoup injuries and microlesions.
- These findings were detected using 3-T and 7-T MRI but their detection and assessment are hampered at 3 T.
- 7-T brain MRI could be able to determine the absence of shearing brain injuries.

## Background

The term “virtual autopsy” describes the use of imaging technologies as a supplement or alternative to traditional autopsy. For this approach, postmortem magnetic resonance imaging (MRI) has been applied for the radiologic assessment of cerebral tissue [1]. The detection and assessment of traumatic brain injuries and small cerebral lesions can be of particular interest in forensic investigations for differentiating between traumatic and nontraumatic origins of cerebral haemorrhages.

During a traumatic event, the application of different forces to the head can result in characteristic alterations such as shearing or coup and contrecoup injuries [2, 3]. Shearing injuries accrue via acceleration-deceleration or rotational acceleration mechanisms of force, resulting in axonal damage [4, 5]. Coup/contrecoup injuries describe tissue damaged by bruising of the brain directly related to the trauma at the site of impact (coup injury) or at the opposite side when the brain hits the skull (contrecoup injury) [6]. The identification of coup/contrecoup injuries can provide information about the direction of the traumatic impact. Contrecoup injuries are usually more pronounced than coup injuries and occur on the opposite side of the impact [6]. In contrast, nontraumatic haemorrhages are usually caused by underlying diseases, such as hypertension or amyloidosis, which are frequently accompanied by microhaemorrhages (also referred to as microbleeds) and small ischaemic lesions (microinfarcts) [7, 8]. The detection of such microlesions using MRI is highly dependent on the spatial resolution of the images; consequently, the sensitivity for microlesions was first increased by switching from 1.5-T to 3-T [9] and from 3-T to 7-T MRI [10]. However, a higher magnetic field strength is unfortunately also accompanied by increased inhomogeneities in the main magnetic field (*B*_0_) as well as in the transmit and receive fields (*B*_1_) [11]. These inhomogeneities can negatively affect the image signal and contrast, especially at the periphery of the measured object.

Considering the benefits and drawbacks of 7-T over 1.5-T and 3-T MRI, we deemed it relevant to assess the application of postmortem 7-T MRI for the assessment of traumatic and nontraumatic origins of cerebral haemorrhages. To date, the use of noninvasive postmortem 7-T MRI has only been evaluated for fetal virtual autopsy or radiologic wound ballistics in decedents [12, 13]. In this article, we present two decedents with cerebral haemorrhages due to a traumatic event or a natural cause who underwent noninvasive postmortem computed tomography (CT), 3-T, and 7-T MRI for forensic purposes. The focus of this article is the detectability and assessability of coup/contrecoup injuries and microlesions.

## Methods

The responsible ethics committee waived ethical approval, as all examinations were performed as part of forensic judicial investigations.

The first case was a 92-year-old woman who was found at the bottom of a staircase. The second case was a 68-year-old man who was found in his bathroom lying on the floor. The postmortem intervals were 14-16 h (case 1) and 26-30 h (case 2) when postmortem imaging was performed. Before MRI, the decedents underwent a routine CT examination using a 128-slice scanner (Somatom Definition Flash, Siemens Healtheeners, Erlangen, Germany) [14]. The parameters of the postmortem head and neck scan were as follows: tube voltage 120 kVp; tube current 1,000 mAs; slice thickness 0.6 mm; kernels H60 and H31; and field of view ≤ 300 mm, adjusted to the head.

After the CT scan, the decedents were examined using a 3-T MRI scanner (Achieva 3.0 T TX, Philips Healthcare, Best, The Netherlands) and a 7-T MRI scanner (Magnetom Terra, Siemens Healthneers, Erlangen, Germany). For comparison with CT, a 7-T three-dimensional gradient-echo sequence (repetition time 4.2 ms, echo time 1.5 ms, voxel size 0.20 mm × 0.20 mm × 0.50 mm, scan time 6:21 min:s) was performed to visualise any osseous injuries. For the comparison of coup/contrecoup injuries and microlesions detected at 3-T and 7-T MRI, T1-weighted, T2-weighted, and susceptibility-weighted sequences were obtained (Table 1). Imaging was performed while the decedents were lying in the body bag.

**Table 1:** 3-T and 7-T protocols and radiologic assessment of coup/contrecoup injuries and microlesions

Sequence	3 T	7 T	3 T	7 T	3 T	7 T
	T1-w. TFE	T1-w. MP2RAGE	T2-w. TSE SPAIR	T2-w. SPACE	VenBOLD	SWI
Repetition time (ms)	9.4	4,500	1,500	4,000	18	21
Echo time (ms)	4.6	2	230	118	26	14
Voxel size*	1.00 × 1.00 × 2.00	0.65 × 0.65 × 0.63	0.80 × 0.80 × 1.60	0.71 × 0.71 × 0.67	0.80 × 0.80 × 1.60	0.12 × 0.12 × 1.50
Slice orientation	Sagittal	Sagittal	Transversal	Sagittal	Transversal	Transversal
Scan time (min:s)	08:50	10:45	06:00	05:39	06:21	10:40
Coup injury	1−	2+	1+	3++	1+	3++
Contercoup injury	1−	2+	1+	3++	1+	3++
Microinfarct	1−	2−	1+	2++	1-	3-
Microhaemorrhage	1−	2−	1+	2++	1+	3+

*MP2RAGE* Magnetisation prepared 2 rapid gradient echoes, *SPACE* Sampling perfection with application-optimised contrasts by using different flip angle evolutions, *SPAIR* Spectral attenuated inversion recovery, *SWI* Susceptibility-weighted imaging, *TFE* Turbo field echo, *TSE* Turbo spin echo, *VenBOLD* Venous blood oxygen level dependent

*Frequency direction × phase direction × slice thickness

The assessability (related to the image quality) and the detectability of the coup/contrecoup injuries and microlesions were graded using 3-point Likert scales. For assessability, images were assigned score 1 (poor image quality, blurred images), score 2 (moderate image quality), or score 3 (good image quality, sharp images). For lesion detectability, images were scored “−” (lesions not detected or inconclusive), “+” (lesions detected), or “++” (lesions highlighted or more accurately depicted than on comparative images). Grading was conducted by a board-certified radiologist with 13 years of professional experience.

## Results

The grading of the coup/contrecoup injuries and microlesions are summarised in Table 1.

### Case 1 (traumatic injuries)

**Fig. 1:**
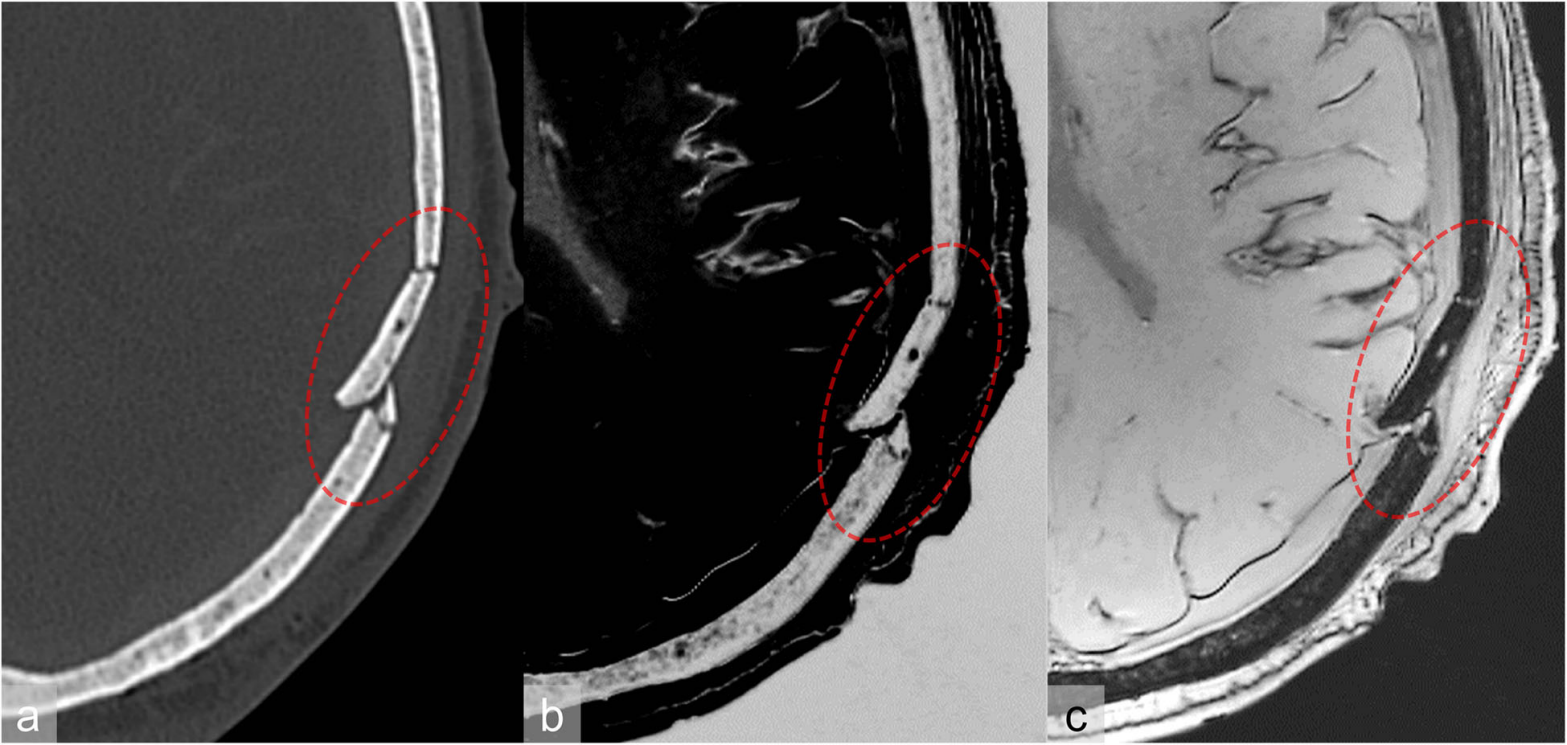
Case 1. Computed tomography (**a**) and 7-T magnetic resonance imaging (**b**: spoiled gradient-echo sequence, contrast inverted; **c**: spoiled gradient-echo sequence) demonstrate a fracture (red circle) of the left parietal bone. The fracture was very well delineated thanks to the high spatial resolution of the 7-T spoiled gradient-echo sequence

On CT and MRI, the skull showed a disruption of the surface and subcutaneous haemorrhages and a segmental bone fracture of the left parietal bone, which was identified as the site of the impact (Fig. 1). Furthermore, subarachnoid, subdural, and brainstem haemorrhages were visible on CT as well as on MRI.

**Fig. 2:**
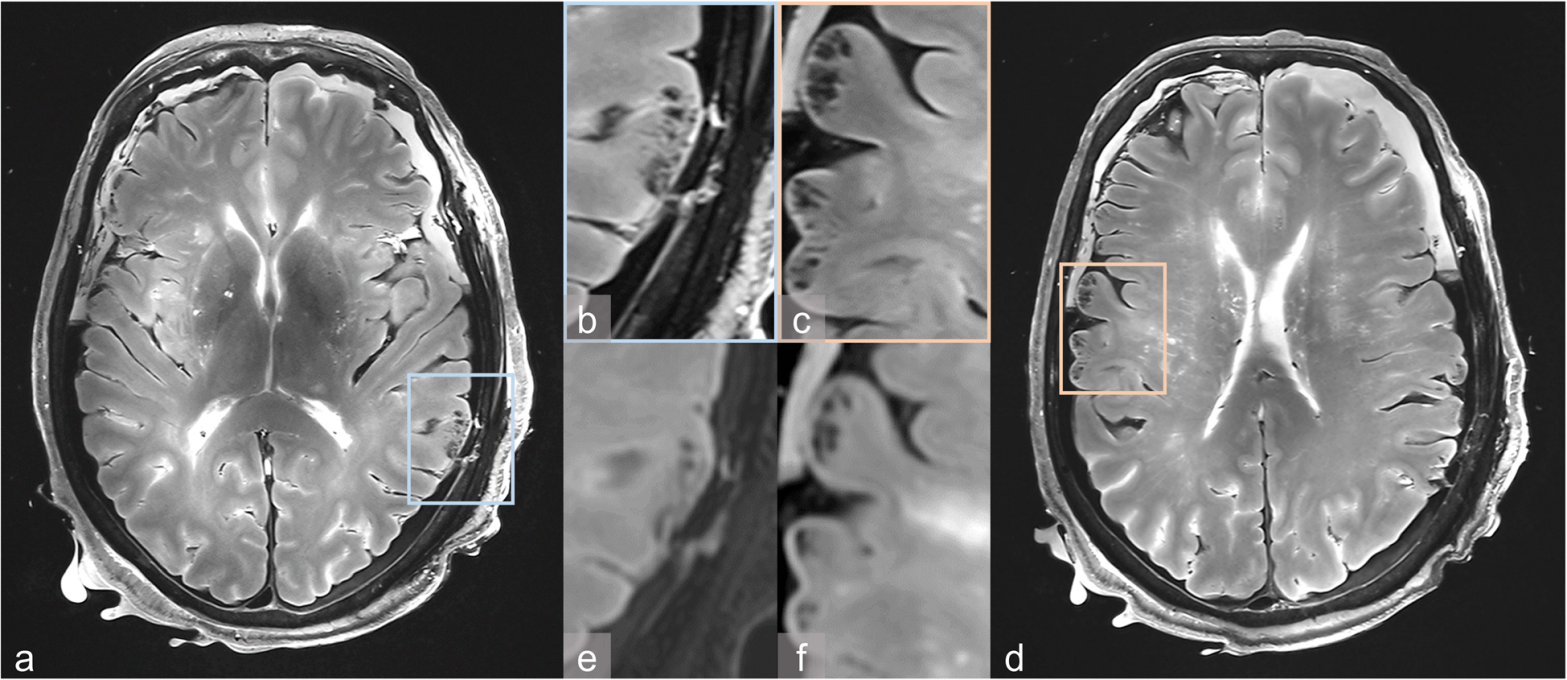
**a-d** Case 1. 7-T T2-weighted images demonstrate coup injuries (**a**, **b**) and contrecoup injuries (**c**, **d**). 3-T T2-weighted images appear blurred for both coup injuries (**e**) and contrecoup injuries (**f**)

Only MRI allowed the detection of discrete parenchymal defects in the left parietal and temporal cortex, which were described as coup injuries. Corresponding contrecoup injuries were detected on the opposite side in the right parietal and temporal cortex on MRI (Fig. 2). These subtle parenchymal defects with intraparenchymal haemorrhages were < 2 mm in size. The coup/contrecoup injuries were highlighted on 7-T MRI using the T2-weighted sequence and the susceptibility-weighted sequence. The clear delineation of the subtle parenchymal defects on 7-T MRI facilitated the identification of the contrecoup injuries and enabled us to exclude the presence of shearing injuries, as no shear strain or signs of physical disruptions along the white and grey matter junction were detected. On 3-T MRI, the subtle parenchymal defects appeared blurrier than those on 7-T MRI (Fig. 2), and shearing injuries could not be excluded.

The radiologic findings were confirmed by autopsy. The cause of death was attributed to a brainstem haemorrhage. The manner of death was determined to be unnatural (accidental death) because of the parietal bone fracture and coup and contrecoup injuries.

### Case 2 (nontraumatic injuries)

**Fig. 3:**
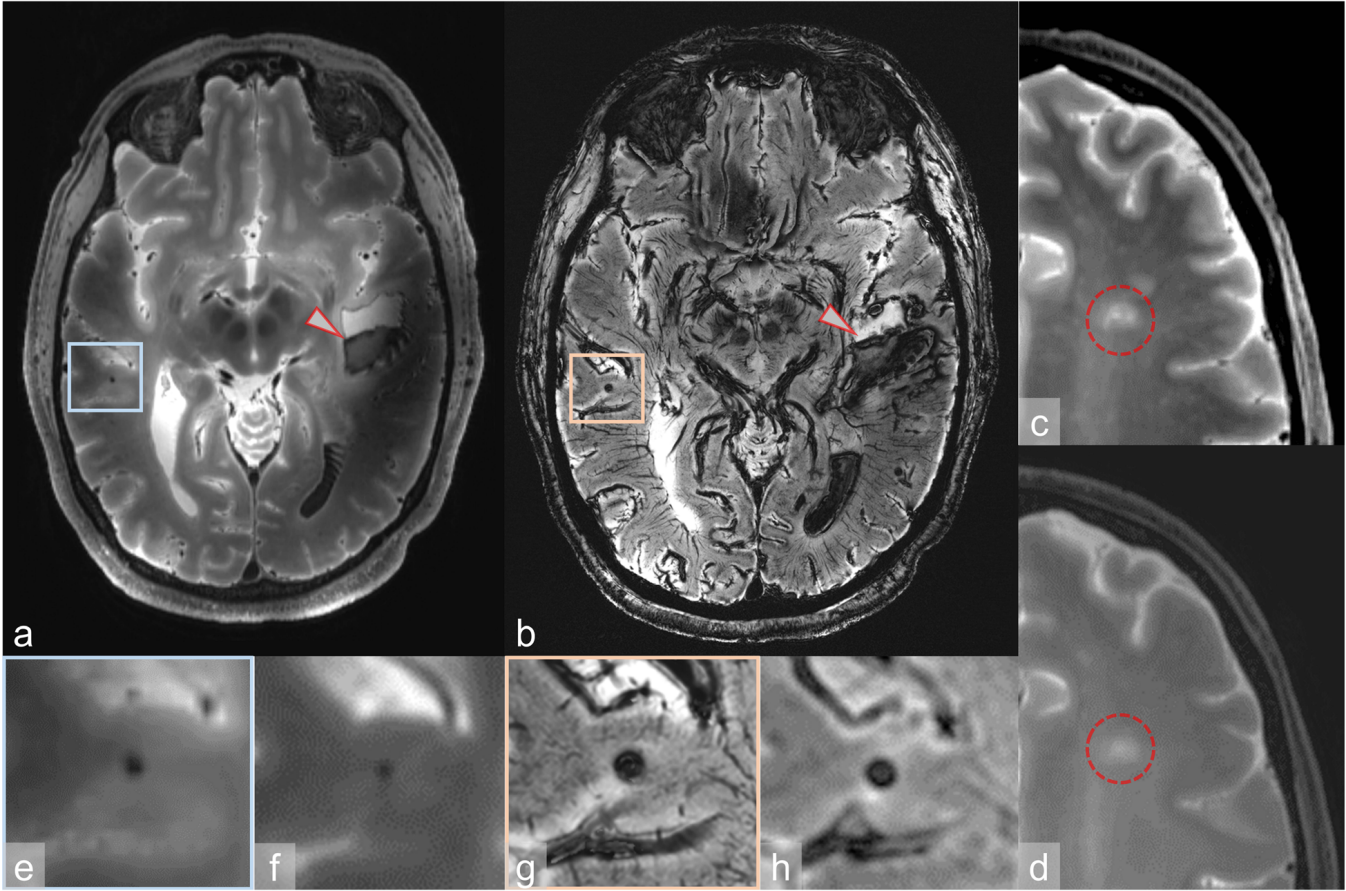
**a-h** Case 2. An intraparenchymal haemorrhage was delineated on 7-T MRI (arrowhead in **a**: T2-weighted sequence (sampling perfection with application-optimised contrasts by using different flip angle evolutions, SPACE) and in **b**: susceptibility-weighted imaging). The haemorrhage presented with postmortem sedimentation. The SPACE image is limited by signal inhomogeneity visible on the anatomically left side adjacent to the haemorrhage. In addition, MRI detected subtle lesions, better delineated at 7 T than at 3 T: microinfarcts (**c**: 7-T T2-weighted SPACE sequence; **d**: 3-T T2-weighted turbo field echo sequence), and microhaemorrhages (**e** and **g** 7-T T2-weighted SPACE sequence and 7-T susceptibility-weighted imaging; **f** and **h**, 3-T T2-weighted turbo field echo sequence and venous blood oxygen level-dependent imaging)

An intracerebral haemorrhage in the left temporal lobe was diagnosed at CT and MRI (Fig. 3a, b). The assessment of the surrounding tissue was impeded on CT scans by an accompanying brain edema. On MRI, the haemorrhage was clearly delineated, although the 3-T images appeared blurrier than the 7-T images. The 7-T T2-weighted images, however, demonstrated visible signal inhomogeneity on the lateral left side adjacent to the haemorrhage (Fig. 3a).

Only MRI allowed the detection of a small T2-hyperintense ischaemic microinfarct next to the head of the caudate nucleus in the left hemisphere (Fig. 3c, d). On 7-T T2-weighted images, this white matter lesion was delineated with moderate image quality compared with the blurriness of the 3-T images. The diameter of the microinfarct differed between 3-T and 7-T T2-weighted images: the maximum diameter was 8.2 mm at 3 T and 9.6 mm at 7 T. The same applied for multiple microhaemorrhages (diameter < 1 mm) that were located in the right parietal and temporal lobe areas, which were visualised smaller on 3-T than on 7-T T2-weighted images (Fig. 3e, f). These differences in size were not observed on the susceptibility-weighted images. However, microhaemorrhages appeared darker on the sharp 7-T images than on the blurry 3-T images (Fig. 3g, h).

The detection of ischaemic microinfarcts and multiple microhaemorrhages, together with the absence of bone fractures, bone edema, shearing injuries, or coup/contrecoup injuries, allowed the determination of the large intracerebral haemorrhage as a naturally caused injury. Accordingly, the manner of death was determined to be natural; therefore, the state attorney waived the need for an additional autopsy.

## Discussion

The MRI examinations enabled the visualisation of coup and contrecoup injuries (case 1) and microlesions (case 2) that were not identifiable on CT scans. Compared with 3-T MRI, 7-T MRI improved the assessability of the extent of the coup and contrecoup injuries and facilitated the detection of microlesions.

Overall, 3-T images appeared blurrier than the 7-T images, as expected. In fact, spin polarisation is increased for 7-T units, compared with that of the widely used 1.5-T and 3-T units, which in turn leads to a stronger signal and thus an increased signal-to-noise ratio [15]. The increased signal-to-noise ratio directly improves the image quality and can be used to improve spatial resolution or to shorten the scan time for the same spatial resolution [16]. Due to the roughly twofold lower signal-to-noise ratio at 3-T than at 7-T, the scan time would need to be at least four times longer to achieve similar image quality on 3-T MRI with the same spatial resolution. A drawback of 7-T MRI is the greater signal inhomogeneity due to the transmit B_1_ inhomogeneity. This phenomenon was illustrated by case 2. To improve the image quality in the presence of inhomogeneous magnetic fields, various approaches have been investigated regarding acquisition (*e.g.,* the use of multichannel transmit arrays [17, 18]) or postprocessing (*e.g.,* improved image reconstruction algorithms [19]).

The use of sharp thin-sliced 7-T images with high sensitivity to metals is appreciated for the radiologic diagnosis of subtle lesions and iron accumulation in specific brain diseases [20–24]. Especially for the early diagnosis of multiple sclerosis, 7-T MRI can be of particular importance, since 7-T MRI improves the ability to detect smaller and earlier multiple sclerosis lesions and 7-T MRI allows a more accurate characterisation of these lesions for discriminating multiple sclerosis from other brain diseases compared to 1.5-T and 3-T MRI [25].

According to our observations, 7-T MRI can facilitate the classification of the origin of cerebral haemorrhages as either traumatic or natural. In case 1, the detection of coup/contrecoup injuries allowed the description of the impact, leading to the diagnosis of an acceleration-deceleration injury. The exclusion of shearing injuries was facilitated on 7-T images, which can be considered an advantage over 3-T MRI and may be of special value in the assessment of child abuse [26]. The use of a three-dimensional gradient-echo sequence at 7 T provided a detailed visualisation of the parietal bone fracture. As a consequence, this approach may be appropriate for delineating cranial fractures on MRI. For the same purpose, dedicated sequences can be applied at 3 T [27, 28]. An accurate depiction of cranial fractures on MRI can aid in the assessment of related soft tissue injuries.

In case 2, 7-T MRI showed a slight advantage over 3-T MRI in the classification of discrete lesions, in accordance with previous research [29]. The 7-T T2-weighted images not only allowed a clear distinction of the microinfarct from the surrounding tissue but also depicted the rather precise extent of this microlesion, which is due to the small isotropic voxels of the 7-T sequence. On T2-weighted images, the microhaemorrhages were larger in diameter at 7 T than at 3 T, in accordance with the literature [30, 31]. On susceptibility-weighted images, microhaemorrhages were highlighted at both 3 T and 7 T, due to susceptibility effects of paramagnetic deoxygenated blood [32]. A voxel size of 1.5 mm (7-T MRI) or 1.6 mm (3-T MRI) in the slice direction still enabled the clear visualisation of these subtle lesions on susceptibility-weighted images. An increase in the diameter of the microhaemorrhages at 7 T over 3 T, as observed on the T2-weighted images, was not observed on the susceptibility-weighted images. Likewise, only a small increase in the diameter of the microhaemorrhages has been reported between 1.5-T and 3-T MRI in the literature [33].

In conclusion, all distinctive and subtle findings were detectable on both 3-T and 7-T MRI, but the 7-T images provided higher detectability and assessability of traumatic brain injuries and microlesions. Certainly, the relevance of this advantage should be weighed against the practicability of 7-T MRI, which is dependent on several factors, in particular the accessibility to a 7-T scanner. Notwithstanding this limitation, further studies on 7-T MRI for various diagnostic purposes are appreciated.

## Abbreviations

CT: Computed tomography
MRI: Magnetic resonance imaging
SPACE: Sampling perfection with application-optimised contrasts by using different flip angle evolutions

## References

[1] Thali MJ, Yen K, Schweitzer W (2003). Virtopsy, a new imaging horizon in forensic pathology: virtual autopsy by postmortem multislice computed tomography (MSCT) and magnetic resonance imaging (MRI)—a feasibility study. J Forensic Sci.

[2] Courville CB (1942) Coup-contrecoup mechanism of craniocerebral injuries: some observations. Arch Surg 45:19–43. 10.1001/archsurg.1942.01220010022002

[3] Peerless SJ, Rewcastle NB (1967). Shear injuries of the brain. Can Med Assoc J.

[4] Strich SJ (1956) Diffuse degeneration of the cerebral white matter in severe dementia following head injury. J Neurol Neurosurg Psychiatry 19:163–185. 10.1136/jnnp.19.3.163 10.1136/jnnp.19.3.163 PMC497203 13357957

[5] Rush B, Kreutzer JS, DeLuca J, Caplan B (2011). Shearing injury, shear strain. Encyclopedia of Clinical Neuropsychology.

[6] Bauer M, Polzin S, Patzelt D (2004). The use of clinical CCT images in the forensic examination of closed head injuries. J Clin Forensic Med.

[7] Viswanathan A, Chabriat H (2006). Cerebral microhemorrhage. Stroke.

[8] Van Veluw SJ, Jolink WM, Hendrikse J (2014). Cortical microinfarcts on 7 T MRI in patients with spontaneous intracerebral hemorrhage. J Cereb Blood Flow Metab.

[9] Scheid R, Ott DV, Roth H, Schroeter ML, von Cramon DY (2007). Comparative magnetic resonance imaging at 1.5 and 3 Tesla for the evaluation of traumatic microbleeds. J Neurotrauma.

[10] Bian W, Hess CP, Chang SM, Nelson SJ, Lupo JM (2014). Susceptibility-weighted MR imaging of radiation therapy-induced cerebral microbleeds in patients with glioma: a comparison between 3 T and 7 T. Neuroradiology.

[11] Uwano I, Kudo K, Yamashita F (2014). Intensity inhomogeneity correction for magnetic resonance imaging of human brain at 7 T. Med Phys.

[12] Staicu A, Albu C, Popa-Stanila R (2019). Potential clinical benefits and limitations of fetal virtopsy using high-field MRI at 7 Tesla versus stereomicroscopic autopsy to assess first trimester fetuses. Prenat Diagn.

[13] Gascho D, Deininger-Czermak E, Zoelch N et al (2020) Noninvasive 7 tesla MRI of fatal craniocerebral gunshots – a glance into the future of radiologic wound ballistics. Forensic Sci Med Pathol. 10.1007/s12024-020-00300-w 10.1007/s12024-020-00300-w PMC7669810 32920765

[14] Gascho D, Thali MJ, Niemann T (2018). Post-mortem computed tomography: technical principles and recommended parameter settings for high-resolution imaging. Med Sci Law.

[15] Pohmann R, Speck O, Scheffler K (2016). Signal-to-noise ratio and MR tissue parameters in human brain imaging at 3, 7, and 9.4 tesla using current receive coil arrays: SNR at 9.4 T. Magn Reson Med.

[16] Wald LL (2012). The future of acquisition speed, coverage, sensitivity, and resolution. Neuroimage.

[17] Orzada S, Bitz AK, Johst S (2017). Analysis of an integrated 8-channel Tx/Rx body array for use as a body coil in 7-Tesla MRI. Front Phys.

[18] Kraff O, Fischer A, Nagel AM, Mönninghoff C, Ladd ME (2015). MRI at 7 tesla and above: demonstrated and potential capabilities. J Magn Reson Imaging.

[19] George MM, Kalaivani S (2019). Retrospective correction of intensity inhomogeneity with sparsity constraints in transform-domain: application to brain MRI. Magn Reson Imaging.

[20] Hammond KE, Metcalf M, Carvajal L (2008). Quantitative in vivo magnetic resonance imaging of multiple sclerosis at 7 Tesla with sensitivity to iron. Ann Neurol.

[21] Tallantyre EC, Morgan PS, Dixon JE (2010). 3 Tesla and 7 Tesla MRI of multiple sclerosis cortical lesions. J Magn Reson Imaging.

[22] Kwan JY, Jeong SY, Gelderen PV (2012). Iron accumulation in deep cortical layers accounts for MRI signal abnormalities in ALS: correlating 7 Tesla MRI and pathology. PLos One.

[23] Dusek P, Bahn E, Litwin T (2017). Brain iron accumulation in Wilson disease: a post mortem 7 Tesla MRI – histopathological study. Neuropathol Appl Neurobiol.

[24] Cocozza S, Cosottini M, Signori A (2020). A clinically feasible 7-Tesla protocol for the identification of cortical lesions in multiple sclerosis. Eur Radiol.

[25] Bruschi N, Boffa G, Inglese M (2020). Ultra-high-field 7-T MRI in multiple sclerosis and other demyelinating diseases: from pathology to clinical practice. Eur Radiol Exp.

[26] Hart BL, Dudley MH, Zumwalt RE (1996). Postmortem cranial MRI and autopsy correlation in suspected child abuse. Am J Forensic Med Pathol.

[27] Eley KA, Watt-Smith SR, Golding SJ (2012). “Black bone” MRI: a potential alternative to CT when imaging the head and neck: report of eight clinical cases and review of the Oxford experience. Br J Radiol.

[28] Gascho D, Zoelch N, Tappero C (2020). FRACTURE MRI: optimized 3D multi-echo in-phase sequence for bone damage assessment in craniocerebral gunshot injuries. Diagn Interv Imaging..

[29] Springer E, Dymerska B, Cardoso PL et al (2016) Comparison of routine brain imaging at 3 T and 7 T. Invest Radiol 51:469–482. 10.1097/RLI.0000000000000256 10.1097/RLI.0000000000000256 PMC5704893 26863580

[30] Smith EE, Schneider JA, Wardlaw JM, Greenberg SM (2012). Cerebral microinfarcts: the invisible lesions. Lancet Neurol.

[31] Mulder MJ, Keuken MC, Bazin P-L, Alkemade A, Forstmann BU (2019). Size and shape matter: the impact of voxel geometry on the identification of small nuclei. PLoS One.

[32] Liu C, Li W, Tong KA, Yeom KW, Kuzminski S (2015). Susceptibility-weighted imaging and quantitative susceptibility mapping in the brain. J Magn Reson Imaging.

[33] Nandigam RNK, Viswanathan A, Delgado P (2009). MR imaging detection of cerebral microbleeds: effect of susceptibility-weighted imaging, section thickness, and field strength. AJNR Am J Neuroradiol.

